# Carboxypeptidase U (CPU, TAFIa, CPB2) in Thromboembolic Disease: What Do We Know Three Decades after Its Discovery?

**DOI:** 10.3390/ijms22020883

**Published:** 2021-01-17

**Authors:** Karen Claesen, Joachim C. Mertens, Dorien Leenaerts, Dirk Hendriks

**Affiliations:** Laboratory of Medical Biochemistry, Department of Pharmaceutical Sciences, University of Antwerp, 2610 Wilrijk, Belgium; karen.claesen@uantwerpen.be (K.C.); mertens.joachim@gmail.com (J.C.M.); dorien.leenaerts@gmail.com (D.L.)

**Keywords:** arterial thrombosis, carboxypeptidase B2, carboxypeptidase U, fibrinolysis, thrombin-activatable fibrinolysis inhibitor, thromboembolic diseases, venous thrombosis

## Abstract

Procarboxypeptidase U (proCPU, TAFI, proCPB2) is a basic carboxypeptidase zymogen that is converted by thrombin(-thrombomodulin) or plasmin into the active carboxypeptidase U (CPU, TAFIa, CPB2), a potent attenuator of fibrinolysis. As CPU forms a molecular link between coagulation and fibrinolysis, the development of CPU inhibitors as profibrinolytic agents constitutes an attractive new concept to improve endogenous fibrinolysis or to increase the efficacy of thrombolytic therapy in thromboembolic diseases. Furthermore, extensive research has been conducted on the in vivo role of CPU in (the acute phase of) thromboembolic disease, as well as on the hypothesis that high proCPU levels and the Thr/Ile325 polymorphism may cause a thrombotic predisposition. In this paper, an overview is given of the methods available for measuring proCPU, CPU, and inactivated CPU (CPUi), together with a summary of the clinical data generated so far, ranging from the current knowledge on proCPU concentrations and polymorphisms as potential thromboembolic risk factors to the positioning of different CPU forms (proCPU, CPU, and CPUi) as diagnostic markers for thromboembolic disease, and the potential benefit of pharmacological inhibition of the CPU pathway.

## 1. Introduction

The basic carboxypeptidase zymogen procarboxypeptidase U (proCPU), also known as thrombin-activatable fibrinolysis inhibitor (TAFI), procarboxypeptidase R (proCPR) or plasma procarboxypeptidase B (proCPB), is predominantly synthesized by the liver and secreted into plasma [1,2,3,4,5]. Upon activation by thrombin(-thrombomodulin) or plasmin, the key-enzymes of the coagulation and fibrinolysis respectively, the zymogen is converted into a potent attenuator of fibrinolysis: carboxypeptidase U (CPU, TAFIa, CPB2) [5,6,7]. CPU counteracts progressing plasminogen activation by cleaving off C-terminal lysine residues from partially degraded fibrin, which are essential for effective clot dissolution, thereby delaying fibrinolysis [5]. Rapid thermal inactivation through a conformational change (t_1/2_ = 8–15 min, depending on the +1040C/T polymorphism) and further proteolytic cleavage will subsequently convert CPU to an inactive form (CPUi), making the effect of CPU self-limiting [5,8]. Interestingly, CPU exerts its activity in a threshold-dependent manner: as long as the CPU level remains above a certain threshold value fibrinolysis is halted, but once the CPU activity drops below this level, plasmin formation is facilitated and the fibrinolysis will accelerate. Since CPU is considered as a prominent bridging molecule between coagulation and fibrinolysis, the development of CPU inhibitors as profibrinolytic agents constitutes an attractive new concept to improve endogenous fibrinolysis (thereby preventing thrombosis) or to increase the efficacy of thrombolytic therapy. Furthermore, extensive research has been conducted on the in vivo role of CPU in (the acute phase of) thromboembolic disease, as well as on the hypothesis that high proCPU levels or specific *CPB2* gene variants may cause a thrombotic predisposition. This review provides an overview of the methods available for measuring proCPU, CPU and CPUi, together with a summary of the clinical data generated so far, ranging from the current knowledge on proCPU concentrations and polymorphisms as potential thromboembolic risk factors, to the positioning of different CPU forms (proCPU, CPU and CPUi) as diagnostic markers for thromboembolic disease, and the potential benefit of pharmacological inhibition of the CPU pathway.

## 2. Measurement of ProCPU, CPU and CPUi: Methods, Challenges and Pitfalls

**Figure 1:**
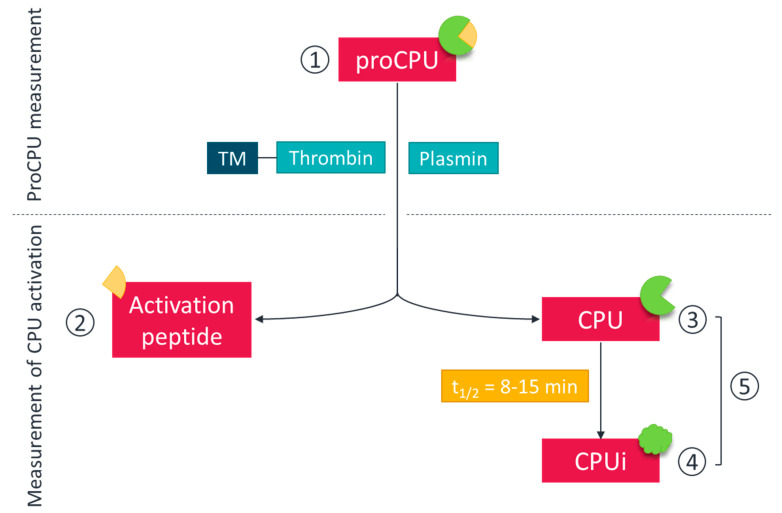
Measurement of different forms of the *CPB2* gene product. The zymogen procarboxypeptidase U (proCPU) (**1**) can be measured with antigen- or activity-based assays. Carboxypeptidase U (CPU) activation can also be determined. Upon activation with thrombin, the thrombin-thrombomodulin (TM) complex, or plasmin, the activation peptide (AP; **2**) is released and can be measured with an antigen-based assay. The active enzyme CPU can be directly measured by enzymatic assays (**3**). The active enzyme CPU is thermally inactivated into CPUi (**4**) which can be measured with antigen-based assays that detect both CPU and CPUi simultaneously (**5**).

To date, a wide range of methods is available for the quantification of proCPU, CPU and CPUi, including immunological (antigen-based) methods, enzymological (activity-based) methods, and functional fibrinolytic assays (Figure 1). Each method is accompanied by inherent advantages and shortcomings, so it is of utmost importance to carefully select an appropriate, well-characterized, and validated assay when investigating the (patho)physiological role of specific CPU forms (proCPU, CPU, and CPUi).

### 2.1. ProCPU

Various in-house developed assays and commercially available quantification kits exist for the determination of proCPU in plasma (Table 1) [9,10,11,12,13]. Immunoassays (enzyme-linked immunosorbent assays, ELISAs) are relatively easy to perform, can be automated efficiently, do not require activation of the zymogen prior to measurement, and are not comprised by interference of plasma carboxypeptidase N (CPN) [14]. A major challenge with this type of assays is cross-reactivity of the antibodies raised against proCPU with other components, such as CPU, inactivated CPU (CPUi), the activation peptide or other proteolytic fragments [12]. Also, depending on the antibodies used, ELISAs lack interspecies cross-reactivity which requires the necessity of species-specific antibody combinations and hampers flexibility towards animal studies [14,15]. Another issue is the unequal reactivity of certain ELISAs towards different proCPU isoforms of the Thr/Ile325 polymorphism, resulting in incorrect proCPU measurement [12,16,17]. In a comparative study by Heylen et al., three commercially available antigen-based assays (Zymutest^®^ TAFI, Visulize^®^ TAFI and Immunoclone^®^ TAFI) showed significantly lower reactivity towards the Ile325-isoform, giving rise to an overestimation of the variation between the different genotypes [18]. As these assays have been applied in clinical studies, the reported results need to be reinterpreted in this light [18]. Nevertheless, these three assays are still marketed today, without mentioning the difference in reactivity towards the Thr/Ile325 isoforms. Several other suppliers offer proCPU ELISA kits, but their ability to distinguish between proCPU, CPU, and CPUi, as well as their reactivity to the proCPU polymorphisms need to be validated before these kits can be reliably implemented in observational and clinical trials.

Alternatively, activity-based enzymological methods can be implemented to quantify proCPU levels (Table 1). These methods require exogenous and quantitative activation of proCPU to CPU by thrombin-thrombomodulin, followed by quantification of the formed active CPU. The latter can be done by means of incubation with C-terminal Arg- or Lys-containing CPU specific substrates. The released Arg/Lys or the other fragment can thereafter be detected via different analytical approaches (HPLC-UV, spectrophotometer or fluorimeter). Unfortunately, most of these substrates (e.g., hippuryl-L-arginine (Hip-Arg or Bz-Gly-Arg) and anisolylazoformylarginine (AAFR)) are not optimal for use in plasma or whole blood samples due to the interfering activity of endogenous CPN [19,20,21]. However, the more recently designed synthetic substrate N-benzoyl-*ortho*-cyano-phenylalanyl-arginine (Bz-*o*-cyano-Phe-Arg) has improved selectivity for CPU over CPN and allowed the development of an assay that does not suffer from interference by CPN [9,22]. Furthermore, proCPU itself has been described to show limited intrinsic enzymatic activity towards these small synthetic substrates, but this does not interfere with the above described proCPU assays, nor is it clinically relevant [23,24]. Other components are not detected in activity assays [23,24]. Another advantage of activity-based assays is that these assays are not hampered by different reactivity towards Thr/Ile325 isoforms, at least, when the proCPU activation is performed at 25 °C/room temperature. With activation at 37 °C, the difference in stability of the two isoforms can bias the results [25]. Furthermore, a well-defined incubation interval to guarantee linear substrate conversion is important. At lower substrate concentrations, linear substrate conversion is only observed in a shorter period of time [25].

Given the diversity of the available assays, proCPU research would benefit from international reference material. Therefore, the Scientific and Standardization Committee (SSC) fibrinolysis subcommittee of the International Society of Thrombosis and Haemostasis (ISTH) initiated an international collaborative study to generate the WHO 1^st^ international reference standard for proCPU. The availability of this standard will be an aid for future assay development, allow a more straightforward comparison of study results, and will contribute to the reduction of the reported variability.

### 2.2. CPU

For a long time, measuring active CPU in the blood circulation and identifying pathological conditions in which CPU generation is enhanced, was not possible with the available methods. Activity-based and functional assays were not sensitive and selective enough to measure ultra-low levels of CPU in the presence of high concentrations of proCPU and CPN [14]. ELISAs at hand were unable to distinguish active CPU from CPUi. However, that changed with the advent of the in-house developed methods of Heylen et al. and Kim et al. (Table 1) [91,92]. In the former, the active enzyme is incubated with the substrate Bz-*o*-cyano-Phe-Arg at 25 °C, followed by the UV-detection of the released Bz-*o*-cyano-Phe in an RP-HPLC system [92]. Kim and co-workers on the other hand developed a functional assay in which plasmin-modified fibrin is covalently bound to a quencher molecule and mixed with fluorescein-labeled plasminogen and the plasma sample [91]. Given the fact that CPU cleaves off C-terminal lysine residues from partially degraded fibrin, the rate of fluorescence increase detected by a plate reader reflects the amount of CPU present in the sample. Both the direct enzymatic assay of Heylen et al. and the functional assay of Kim et al. allow high sensitive quantification of CPU levels as low as 0.09 U/L (18 pM) and 12 pM respectively [91,92].

Although the recently developed methods allow the quantification of active CPU, measurement is still is not straightforward. Several preanalytical and analytical difficulties need to be tackled. The first one is the sample collection. Blood sampling tubes intended for CPU measurement need to contain sodium citrate as an anticoagulant together with a thrombin- and/or plasmin inhibitor, such as chlorometyl ketones (e.g., D-phenylalanyl-L-propylarginylchloromethyl ketone (PPACK/FPR-CK)) or d-Val-Phe-Lys chloromethyl ketone (VFK-CK)) and/or aprotinin, to avoid unwanted ex vivo activation of proCPU [99]. Following blood collection, samples should immediately be placed in iced water in order to minimize degradation of the intrinsically unstable CPU [92]. Moreover, centrifugation for plasma isolation needs to be performed at 4 °C and long-term storage at −80 °C is necessary. Additionally, the emergence of hemolysis must be avoided as it was shown that this interferes with the measurement of CPU activity [100]. Furthermore, the short half-life of CPU at 37 °C also implies that the CPU activity measured in plasma is a snapshot that reflects only a limited timeframe. To accurately measure the influence of CPU activity on a pathologic state, multiple sample collections in the acute stage of a disease should be collected. Together these preanalytical and analytical precautions require very strict procedures and make the organization of CPU assessment in clinical studies very demanding.

### 2.3. Assessment of Overall ProCPU Activation

Another strategy is the assessment of the extent of proCPU activation by quantifying cleavage products, such as (i) the amount of activation peptide or (ii) the amount of active and inactive CPU (CPU + CPUi) (Table 1) [12]. Determination of these cleavage products provides insight into the in vivo activation of the proCPU pathway and thus past and ongoing CPU formation. Although it is not possible to determine the fraction of CPU at a specific time point by these methods, consecutive samplings allow to relatively assess the amount of CPU that has been generated between two specific time points and can possibly provide complementary information to activity-based assays in patient populations where fibrinolysis is impaired [36,58,61,62,79]. Antibody pairs for application in sandwich-ELISAs have been developed and validated by Ceresa and co-workers [12]. A first pair developed by this group allows the measurement of the amount of activation peptide that has been released through activation of proCPU (MA-T12D11/MA-T18A8-HRP). A second antibody pair measures the total amount of CPU + CPUi formed (MA-T30E5/MA-T17D7-HRP) [58,59,61,62,101]. Another assay that allows combined measurement of CPU and CPUi was published by Hulme et al. [102]. This assay was marketed as the Imubind^®^ TAFIa/ai ELISA (US20060183172A1 and US7470519B2) but has been discontinued. At this moment, the only commercially available CPU + CPUi ELISA is the Asserachrom^®^ TAFIa/ai ELISA (Diagnostica Stago, Asnières, France). The assay shows equal reactivity towards CPU and CPUi and is not affected by the Thr/Ile325 polymorphism, but enzymatic degradation of CPU/CPUi by thrombin or plasmin can negatively bias the results (in-house unpublished data).

### 2.4. Assessment of CPU by Functional Fibrinolysis Assays

**Figure 2:**
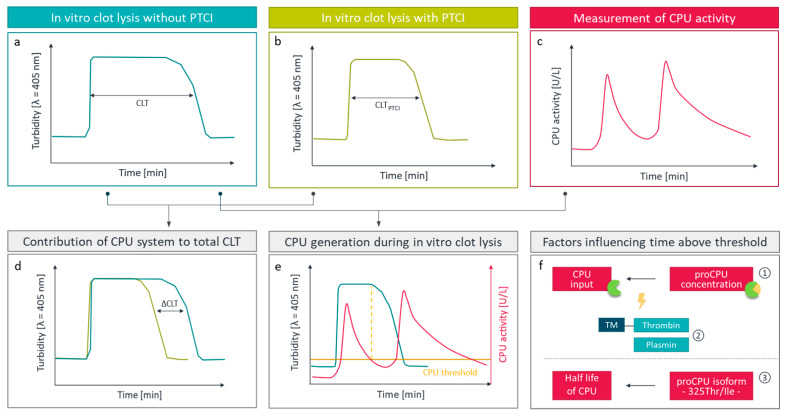
Functional assessment of carboxypeptidase U (CPU). The contribution of CPU to the total clot lysis time (ΔCLT; **d**) can be visualized by means of in vitro clot lysis experiments in the absence (**a**) or presence (**b**) of potato tuber carboxypeptidase inhibitor (PTCI) or a selective CPU inhibitor. During in vitro clot lysis, CPU is generated shortly after initiation of the coagulation—when proCPU is activated by thrombin(-thrombomodulin)—resulting in a first CPU activity peak (**c**) and halting fibrinolysis as long as the CPU level remains above a certain threshold value (**e**). This threshold concentration is dictated by steady-state plasmin concentrations, and thus dependent on tissue-type plasminogen activator (tPA) and plasmin inhibitor concentrations. Once CPU activity falls below this critical threshold value, fibrinolysis accelerates and the generated plasmin gives rise to the formation of a second CPU activity peak (**c**,**e**). The time that the CPU stays above the threshold (**f**) is defined by (**1**) the procarboxypeptidase U (proCPU) concentration, (**2**) the extent of proCPU activation—and thus the concentrations of thrombin(-thrombomodulin)—and (**3**) the CPU half-life, defined by the Thr/Ile325 polymorphism.

It is also possible to use indirect, functional fibrinolytic assays which measure the effect of CPU formation on the fibrinolytic rate. These methods often include experiments with and without the use of a CPU inhibitor, in order to selectively evaluate the contribution of CPU to fibrinolysis (Figure 2). Historically, turbidimetric measurements of clot formation and subsequent lysis in plasma-based systems were applied to functionally assess CPU formation in patients [103]. This type of assay is an interesting and valuable research tool in the characterization of the functional role of CPU and the development of CPU inhibitors but has the downside that it is very sensitive to variations in plasma levels of components of the fibrinolytic and coagulation system. Moreover, various plasma-based systems exist with small variations in the protocol, in terms of activator (Ca^2+^, tissue factor (TF), thrombin, thrombomodulin, phospholipids or combinations of the before mentioned) or tissue-type plasminogen activator (tPA) concentration, that result in high inter-laboratory variability as reported in an international study on the feasibility of a standardized clot lysis assay that was performed by the SSC fibrinolysis subcommittee of the ISTH [104]. Nevertheless, the in-house repeatability of the assays included in this study was excellent [104].

Moreover, combining a classical clot lysis experiment with the measurement of CPU activity on well-defined time points during this clot lysis assay, offers a unique technique to determine an individual’s endogenous CPU generation potential (Figure 2) [105,106,107,108]. It is an interesting tool to study mechanisms that influence CPU generation and may also be valuable to select individuals who would benefit from pharmacological CPU-inhibition [105]. Additionally, Leenaerts et al. demonstrated that the effect of CPU on fibrinolysis can also be assessed in more complex systems. Viscoelastic methods such as thromboelastometry that are performed on whole blood showed a good response to the addition of thrombomodulin (CPU activation) and AZD9684 (CPU inhibition) [94]. In the same study, a complex model of arterial flow has also been assessed but showed substantial variability, thereby limiting its applicability for CPU assessment [94].

## 3. Plasma ProCPU Concentration and ProCPU Polymorphisms as Risk Factors?

Over the years, impaired fibrinolysis has gained attention as a predictor for increased cardiovascular risk [103,109,110]. Given the prominent bridging function of CPU in hemostasis, the question raised whether increased plasma proCPU concentrations could be regarded as a risk factor for thromboembolic diseases. The fact that the Michaelis–Menten constant Km for proCPU activation by thrombin, thrombin-thrombomodulin, or plasmin is considerably above (thrombin and thrombin-thrombomodulin) or within (plasmin) the range of plasma concentrations of proCPU provides a plausible basis for such an association [111,112,113]. When a stimulus is present, the formation of CPU from its precursor proCPU will be directly proportional to the proCPU concentration (Figure 2). In other words, we can hypothesize that a high proCPU concentration might tip the hemostatic balance to antifibrinolytic pathways, causing a predisposition towards a thrombotic tendency [111]. Furthermore, previous research has shown that single nucleotide polymorphisms (SNPs) in the *CPB2* gene contribute to plasma proCPU concentrations, leading to the hypothesis that some of these proCPU variants might also contribute to a higher risk for thromboembolic diseases. Consequently, numerous studies aimed at evaluating the association between proCPU levels and thromboembolic diseases and investigating the role of *CPB2* SNPs as a risk factor. Concerning the latter, this review solely focuses on the +1040C/T polymorphism (corresponding to a Thr/Ile substitution at position 325; Thr/Ile325) as a potential cardiovascular risk factor.

### 3.1. Venous Thrombosis

Studies investigating possible alterations of plasma proCPU levels in different venous thromboembolic diseases (VTE), including deep venous thrombosis (DVT) and pulmonary embolism (PE), are summarized in Table 2 [45,56,77,114,115]. Overall, these studies indicate that high plasma proCPU levels are associated with VTE and can be considered as a mild risk factor (reviewed in [110] and [112]). However, as for the association between the Thr/Ile325 polymorphism and venous thrombosis risk, results have been inconclusive with some studies failing to detect any association and others that found clear correlations. Qian and co-workers performed a meta-analysis on 13 original studies and showed clear evidence that the +1040C/T polymorphism was associated with an increased risk of VTE (cerebral venous thrombosis (CVT), DVT, or PE) [116]. Considering some case-control studies were omitted while others were falsely included, a similar meta-analysis was carried out by Wang et al. taking into account these limitations. In line with the results of Qian et al., the risk to develop venous thrombosis was significantly lower in carriers of the TT genotype of the +1040C/T polymorphism compared to those with the C allele (CC or CT genotype). Subgroup analysis conducted based on ethnicity revealed similar results in different ethnic groups [117]. In accordance, the meta-analysis of Zwingerman and colleagues showed a decreased risk of VTE for the TT genotype versus the CT+CC genotype in both the overall and European study population, although not significant in the overall study population [118].

In conclusion, there is a rationale that high proCPU levels carry a mild risk factor for the development of venous thrombosis. However, there is still a great need for more prospective studies examining the association between the Thr/Ile325 polymorphism and the risk for VTE before a more explicit conclusion can be drawn.

### 3.2. Arterial Thrombosis

In addition to studies investigating the role of proCPU in VTE, even more studies were published on the association between proCPU concentrations or genotype and arterial thrombosis. Table 2 is based upon earlier overviews published by Leurs et al. and Heylen et al., complemented with additional and new reports, and provides an overview of studies investigating proCPU in arterial thrombosis [5,8]. New reports include the 10-year follow up of the SAHLSIS study. In the first SAHLSIS follow-up by Jood et al. in 2012, levels of CPU activation peptide measured in 517 ischemic stroke survivors three months after the index event, but not simultaneously measured proCPU antigen levels, predicted future death and reoccurrence of vascular events (recurrent stroke, transient ischemic attack or coronary event; N = 37) in the first two years [31]. However, at 10-year follow-up, this association could not be confirmed [101]. De Bruijne et al. described an association between proCPU antigen levels and an increased risk of premature peripheral arterial disease [59]. Moreover, Khalifa et al. reported that proCPU levels were not correlated with the pre-disposition of in-stent restenosis following coronary stenting [98]. However, it should be noted that the ELISA used in this last study measured CPU + CPUi levels and not proCPU antigen levels. 

In the last decade, some groups also looked into the association of the +1040C/T polymorphism with increased risk for arterial thrombosis. Rattanawan and co-workers selected a total of 327 patients that were scheduled for an elective coronary angiography and found that the +1040C/T polymorphism was not associated with more severe coronary stenosis [125]. Another study reported that the Thr/Ile325 polymorphism was associated with an increased risk for STEMI but not for idiopathic ischemic stroke in young individuals [124]. A meta-analysis conducted by Shi and colleagues showed that the Thr/Ile325 variant had no significant influence on the susceptibility for cardiovascular diseases (CVD). Moreover, sub-analysis based on ethnicity did not show an association between the polymorphism and CVD as well. However, sub-analysis did reveal that TT (Ile/Ile) genotype carriers had a 25% higher risk of coronary heart disease than those with a TC (Thr/Ile) or CC (Thr/Thr) genotype [134]. In contrast, the results of the meta-analysis of Wang et al. supported the independent association of the +1040C/T polymorphism with the extent and severity of cardiovascular and cerebrovascular diseases, especially among Asian populations and more for the development of cardiovascular, than cerebrovascular diseases [135]. However, it is of note that one of the included studies investigated the association between certain *CPB2* SNPs and CVT risk, whereas all other studies focused on arterial thrombosis or coronary artery disease.

Overall, several studies in which proCPU concentrations were measured before or ample time after the acute phase of arterial thrombosis, indicated that high proCPU levels are associated with a thrombotic tendency, whereas other prospective and retrospective studies were not able to demonstrate an association or even found that high proCPU levels are protective. As a result, data are inconclusive and no consensus was reached so far with regard to proCPU levels and arterial thrombosis. Nevertheless, it must be emphasized that alongside differences in population, risk factors, or definition of control groups, another reason for these discrepancies between results originates from the variety of proCPU assays that were used, which might provide different results depending on the assay type and properties. On the other hand, these conflicting data can also indicate that variations in the proCPU plasma concentration might simply be too small to be regarded as an important risk factor in arterial thrombosis. As a result, measurement of proCPU levels in non-acute settings might not be the most appropriate marker for the investigation of the contribution of the CPU pathway to the development and outcome of thrombotic events. Moreover, the contribution of the Thr/Ile325 polymorphism seems also limited with regard to the risk for arterial thrombosis.

### 3.3. Cardiovascular Risk Factors

Besides evaluating correlations of proCPU levels and genotypes with risk for thromboembolic diseases, there has also been interest in potential associations of proCPU levels with certain cardiovascular risk factors (hypertension, diabetes mellitus, smoking, hyperlipidemia) and whether proCPU levels could be affected by lifestyle modifications or certain pharmacological treatments.

#### 3.3.1. Hypertension

In this regard, Malyszko et al. investigated the effect of hypertension on proCPU levels in a population of renal transplant patients with normal blood pressure or with unregulated blood pressure, despite treatment with an angiotensin converting enzyme (ACE)-inhibitor, beta-blocker or Ca^2+^ blocker. They found that proCPU levels were elevated in the hypertensive cohort and correlated with diastolic blood pressure in these patients [41]. Higher proCPU levels in 58 hypertensive patients compared to 27 controls, were also reported by Ozkan and colleagues [73]. The administration of amlodipine in 31 of these hypertensive patients led to a significant decrease of 7% in proCPU levels after the first month of therapy compared to the initial value, whereas proCPU levels did not change in 27 patients receiving ramipril [73]. In addition, no significant change in proCPU levels was observed in 12 patients with uncontrolled hypertension under an adequate dose of an ACE-inhibitor before and after the addition of an angiotensin-receptor blocker to their regimen [136]. Further, both proCPU antigen and activity levels were higher in hypertensive patients treated with enalapril compared to untreated and betaxolol treated individuals [42]. 

#### 3.3.2. Hyperlipidemia

In a study by Puccetti and co-workers significantly higher proCPU levels were found in hypercholesterolemic patients, but not in patients with hypertriglyceridemia, isolated low high-density lipoprotein cholesterol or mixed hyperlipoproteinemia compared to controls [32]. In accordance, Santamaria and colleagues described lower proCPU levels in non-hypercholesterolemic individuals, although only in women irrespective of their age [131]. Further, Aso et al. showed that low-density lipoprotein cholesterol was an independent determinant of plasma proCPU levels in 105 patients with type 2 diabetes mellitus (T2DM). In addition, subgroup analysis revealed significantly higher plasma proCPU levels in two subgroups: hypercholesterolemic diabetic patients with or without metabolic syndrome [102]. In another study, proCPU levels were reported to be significantly different in dyslipidemic and normolipidemic subjects, with higher values in the dyslipidemic group [137]. Likewise, significantly higher proCPU levels were seen in 44 hypercholesterolemic patients compared to 40 controls. Treatment of these hypercholesterolemic patients with atorvastatin resulted in a significant decrease in proCPU levels [96]. Decreased proCPU levels were also observed in seven CAPD patients after six months of simvastatin therapy, as well as in 35 hyperlipidemic patients treated with simvastatin for eight weeks and in another study in which 126 patients with previous VTE received rosuvastatin for 28 days [49,69,74]. In contrast, 12-week daily treatment with simvastatin did not result in a change in proCPU levels from baseline in a population of hypercholesterolemic T2DM patients treated with metformin (N = 6) or rosiglitazone (N = 3) [98]. In a population of kidney transplant patients, proCPU levels were higher in 12 hyperlipidemic kidney transplant patients than in the normolipidemic subgroup (N = 31). Three months of fluvastatin daily significantly decreased proCPU levels in the hyperlipidemic subpopulation [69]. Furthermore, it was reported that fenofibrate also reduced proCPU levels in patients with metabolic syndrome and dyslipidemia [75].

#### 3.3.3. Diabetes Mellitus

A third cardiovascular risk factor that has been studied with regard to its effect on plasma proCPU concentrations is diabetes mellitus. For this risk factor, plasma proCPU levels were reported to be comparable between type 1 diabetic patients and healthy subjects [43,50]. Likewise, Verkleij and coworkers found similar proCU levels in T2DM patients compared to non-diabetic controls, whereas a tendency of significantly increased plasma proCPU levels in T2DM patients was observed in other studies [55,82,83,84,96]. Additionally, Hori et al. found higher proCPU levels in obese versus non-obese T2DM patients, whereas Yano and coworkers observed in their population lower levels of plasma proCPU in T2DM patients without microalbuminuria compared to those with microalbuminuria [82,83]. Moreover, both Kitagawa et al. and Rigla et al. showed that plasma proCPU levels correlated with hemoglobin A1c (HbA1C) in T2DM patients and therefore suggested that the elevation in proCPU levels in T2DM is related to chronic hyperglycemia [84,96]. In contrast, Verkleij and colleagues reported similar proCPU levels in both tightly (HbA1C < 6%) and poorly (HbA1C > 9%) regulated T2DM patients [54].

Altogether, the literature currently available designates high proCPU levels to appear together with hypertension, hyperlipidemia and T2DM. Interestingly, several studies indicated that normalizing these parameters by pharmacological intervention reduced proCPU levels, whereas others—especially those that investigated the effect of antihypertensive treatment on circulating proCPU levels—presented opposing results.

## 4. Can ProCPU, CPU or CPUi Serve as Diagnostic or Prognostic Biomarkers for Thromboembolic Disease?

Throughout the years, research groups also investigated different forms of *CPB2* gene product (proCPU, CPU and CPUi) in the acute phase of thromboembolic diseases, thereby aiming to elucidate the in vivo role of CPU and the value of different CPU forms (proCPU, CPU and CPUi) as potential diagnostic markers. Importantly, these studies should be clearly distinguished from prospective and retrospective studies that review the contribution of the CPU pathway to the development and outcome of thrombotic events.

### 4.1. Venous Thrombosis

To the best of our knowledge, the role of proCPU and CPU during the acute phase of VTE has only been studied once. In this study, Schroeder et al. measured proCPU antigen levels in 120 patients with suspected PE in blood collected one hour after admission at the hospital [70]. Results showed that proCPU levels were alike in both patients diagnosed with acute PE and those with suspected yet excluded PE. Concomitant DVT in patients with acute PE did also not influence proCPU levels. However, there was a significant increase in proCPU levels in patients with the highest occlusion rates (95–100%) compared to those with non-massive PE, that correlated inversely with D-dimer levels. Although the presence of acute PE is not associated with proCPU antigen levels, the extent of PE might be [70]. Nonetheless, the mechanism leading to higher proCPU levels in patients with high occlusion rates remains unclear.

### 4.2. Arterial Thrombosis

Montaner et al. and Rooth et al. found elevated proCPU antigen levels in ischemic stroke patients within the first 24 h after onset of the symptoms [64,87]. Ribo and co-workers measured proCPU plasma concentrations in patients with acute proximal middle cerebral artery occlusion on admission and before recombinant tPA (rtPA) administration. Results showed that proCPU levels did not differ between patients that recanalized after one hour of rtPA infusion (N = 25) and patients without recanalization (N = 19) [97]. In contrast, during therapeutic thrombolysis of patients with AIS, a 15–20% decrease in proCPU levels was found by Willemse et al., along with a very significant increase of CPU activity in plasma of these patients after thrombolysis with rtPA (N = 4) [99]. In a follow-up study, Brouns et al. could not detect basal CPU levels at admission of patients with AIS (N = 12), but demonstrated that during thrombolytic therapy, the CPU activity increased and relatively high levels of CPU (4.5–10 U/L) were generated in the circulation. In addition, the extent of proCPU activation during thrombolytic therapy was inversely related to efficacy and safety [33]. The decrease in proCPU levels and corresponding CPU generation was observed in rtPA-treated patients in multiple studies, with larger proCPU consumption being associated with a poor outcome [37,44]. In non-thrombolysed patients, significant plasma proCPU activation (reflecting fibrinolytic activity and/or activation of the coagulation cascade) in the first 72 h after ischemic stroke onset was observed [35]. In the same study cohort, increased proCPU levels were measured in the cerebrospinal fluid of stroke patients [36]. More pronounced plasma proCPU consumption and increased proCPU levels in cerebrospinal fluid have been associated with blood-barrier dysfunction, stroke progression and poor outcome [35,36]. Alessi et al. demonstrated that stroke patients at admission had higher CPU + CPUi levels compared to control subjects and that CPU + CPUi levels were associated with stroke severity in non-thrombolysed patients [37]. However, it was not reported whether precautions were taken during sample collection to avoid ex vivo proCPU activation in this study (Table 3) [37,99].

Similar to the findings of Brouns et al. in acute ischemic stroke, in the hyperacute phase of myocardial infarction, Pang and co-workers found significantly lower proCPU levels in 211 patients with acute coronary syndrome compared to 211 controls [138]. Likewise, Leenaerts et al. detected lower proCPU levels in AMI patients compared to controls, indicating ongoing activation of proCPU which was confirmed by CPU activity levels that were higher in patients than in controls. In the same study, increased intra-arterial CPU activity was found near the occlusion site, demonstrating local CPU generation [93]. In contrast, Skeppholm et al. presented higher proCPU levels in ACS patients in the acute phase of the disease with the Asserachrom TAFI-1B1 ELISA, while no differences in proCPU levels (neither activity nor antigen), were found in ACS patients in the study of Cellai et al. [51,89]. Shantsila and colleagues measured increased proCPU antigen levels in STEMI patients during the subacute phase of the myocardial infarction, reaching a maximum seven days after admission, while proCPU antigen levels in non-STEMI patients were higher at admission followed by a gradual decrease over time [88].

The somewhat contradictory proCPU results make it apparent that this parameter—when used as a single denominator—might not be the most appropriate marker for the exploration of the exact role of CPU during the acute phase of thromboembolic diseases. In contrast, the repeated observation of ongoing proCPU activation simultaneous with marked increases in CPU activity during the acute stage, points out that quantification of the extent of proCPU activation, via measurement of the released activation peptide, formation of CPU or consumption of the zymogen—most likely—is a more reliable and precise marker and will provide a better picture of the in vivo role of proCPU, CPU, and CPUi in these diseases.

## 5. Potential Benefit of the Use of CPU Inhibitors—Overview on Inhibitors Anno 2020

So far, no physiological inhibitors of CPU have been described, but several small molecule, peptide, antibody, and nanobody inhibitors were found or designed. CPU activity is inhibited by non-specific inhibitors of the metallopeptidase activity such as the zinc-chelating agents EDTA and 1,10-phenanthroline and reducing agents that reduce the disulfide bridges in the active site of CPU such as 2-mercaptoethanol and dithiothreitol [2,26,90]. The organic arginine analogs MERGETPA (D,L-2-mercaptomethyl-3-guanidinoethylthiopropanoic acid) and GEMSA (guanidinoethyl-mercaptosuccinic acid) and the lysine analog ε-aminocaproic acid (ε-ACA) inhibit several carboxypeptidases including CPU, but they are not selective for CPU over CPN [19,26,140]. Several natural carboxypeptidase inhibitors were discovered, but only potato tuber carboxypeptidase inhibitor (PTCI), leech carboxypeptidase inhibitor (LCI), and tick carboxypeptidase inhibitor (TCI) have been characterized as competitive inhibitors of CPU with a Ki in the nanomolar range [141,142,143,144]. PTCI reversibly inhibits several carboxypeptidases from subfamily A and B, including CPU, but not CPN [141]. As CPU and CPN have several substrates in common, this selectivity is of utmost importance for the clinical application of CPU inhibitors. 

**Figure 3:**
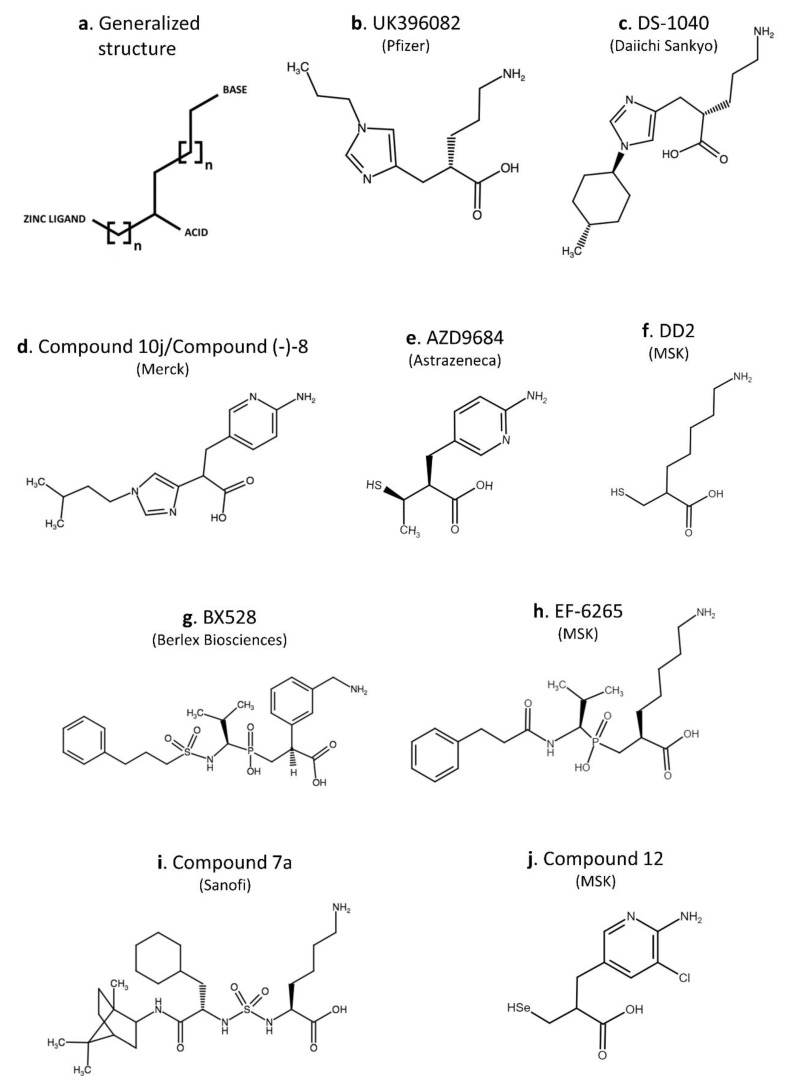
General structure of a low molecular weight (LMW) carboxypeptidase inhibitor (**a**; based on Bunnage et al. [146]) and structures of selective LMW carboxypeptidase U inhibitors disclosed to date (**b**–**j**). Inhibitors containing an imidazole—(**b**–**d**), thiol—(**e**,**f**), dialkyl phosphinic acid—(**g**–**h**), sulfamide—(**i**) or selenium-group (**j**) as the functional group involved in the Zn^2+^-coordination.

Numerous pharmaceutical companies patented a range of low molecular weight (LMW) inhibitors of CPU that are enlisted in Table 4. The generalized structure of an LMW carboxypeptidase inhibitor is depicted in Figure 3 and consists of three characteristic groups that are present in most of the CPU inhibitors: (i) a basic group mimicking the lysine side chain to bind to Asp256, (ii) a carboxylic acid corresponding to the lysine C-terminal carboxylic acid, and (iii) a functional group (carboxylic acid, thiol, imidazole, phosphonic or phosphinic acid, hydroxamic acid, sulfonamide, α-hydroxy ketone, or selenol) to coordinate to the catalytic Zn^2+^ [145]. The clearest categorization of the synthetic CPU inhibitors that have been disclosed to date is by the functional group that is used to coordinate the zinc [146].

Six LMW compounds have reached the clinical development phase: AZD9684 from AstraZeneca (Cambridge, United Kingdom), UK-396082 from Pfizer (New York, USA), SAR104772 and SAR126119 from Sanofi (Paris, France), DS-1040 from Daiichi Sankyo (Tokyo, Japan), and S62798 from Servier (Paris, France).

AZD9684 [(2*S*,3*R*)-2-[(6-aminopyridin-3-yl)methyl]-3-mercaptobutanoic acid] progressed into phase II clinical trials for thrombosis and pulmonary embolism. AZD9684 improved the resolution rate of pulmonary emboli and showed clear stimulation of endogenous fibrinolysis in patients with acute symptomatic embolism, but the development was discontinued in 2007 [148,167]. The compound showed good oral bioavailability (75% in rats), but the short elimination half-life ruled out a once or twice daily administration in patients [149]. The AstraZeneca thiol series which includes AZD9684 was therefore abandoned and novel, conformationally restricted imidazole-based compounds have been developed (4,5,6,7-tetrahydro-1H-benzimidazole-5-carboxylic acid derivatives and 5,6,7,8- tetrahydro-imidazo[1,2α]pyridine-7-carboxylic acid derivatives) [149]. Although the development of AZD9684 has been discontinued, it is still used as a research tool. Using this compound, Leenaerts and co-workers demonstrated that CPU inhibition can stimulate fibrinolysis in several in vitro models with varying levels of complexity [36]. Davidsson and colleagues used AZD9684 to evaluate several biomarkers for the monitoring of fibrinolysis progression [168]. Furthermore, the effect of AZD9684 on outcome parameters and its impact on microvascular thrombosis in a rat transient middle cerebral artery occlusion model has been investigated, showing that selective CPU inhibition resulted in a reduction in fibrin(ogen) deposition and brain edema, which is in its turn suggestive for a reduction in microvascular thrombosis, but without a significant effect on final infarct volume [169].

UK-396082 [(2S)-5-amino-2-[(1-n-propyl-1H-imidazol-4-yl)methyl]pentanoic acid] has a half-life of 4 h after intravenous administration. In addition, oral dosing of this compound revealed good bioavailability. Despite this favorable pharmacokinetic profile, the clinical evaluation of UK-396082 was discontinued by Pfizer in 2011 for unknown reasons [152,170]. Oxygenated derivatives of UK-396082 have been designed by Owen et al. [153], but these compounds did not significantly improve the pharmacology and pharmacokinetics of UK-396082 in rats after IV administration. Similar to AZD9684, also UK-396082 has been used as a research tool after its discontinuation, more specifically in a transgenic mouse model of abdominal aortic aneurysms and in a rat model of kidney fibrosis [63,171,172].

SAR104772—an oral CPU inhibitor from Sanofi—reached phase I in 2010. For SAR126119—a backup compound for SAR104772—phase I was completed in 2011 and phase II was planned to start in 2012 [Sanofi annual report 2011], however, to the best of our knowledge, no data are available from these trials [159]. FFC.HTZ4.059, another CPU inhibitor from Sanofi was evaluated in a murine thromboembolic stroke model. A synergistic effect between FFC.HTZ4.059 and rtPA was observed, but the CPU inhibitor failed to prove superiority over rtPA administration, neither when administered alone nor in combination with a suboptimal dose of rtPA [163]. Sanofi published several patents that disclosed imidazole derivatives as CPU inhibitors (Table 4) and also a patent that disclosed urea and sulfamide derivatives as CPU inhibitors (WO/2008/067909), but the structures of the compounds that were selected for further development have not been disclosed. Recently, it was discovered that anabaenopeptins isolated from cyanobacteria had a CPU inhibitor potency that displayed high selectivity against CPA and CPN [160,161,173]. Starting from these macrocyclic urea derivates, Halland and coworkers started a new lead optimization that resulted in a novel, potent, LMW CPU inhibitor (Compound 3p described in [160]) that in its turn was further developed to a novel sulfamide-containing lead: compound 7a [(S)-6-Amino-2-{[(S)-2-cyclohexyl-1-((1R,2S,4R)-1,7,7-trimethyl-bicyclo[2.2.1]hept-2-ylcarbamoyl)-ethyl-sulfamidyl]}-hexanoic acid] described in [161]). The latter will now be tested in in vivo efficacy studies by Sanofi [161].

DS-1040 [(2S)-5-amino-2-[[1-(4-methylcyclohexyl)imidazol-4-yl]methyl]pentanoic acid] was developed by Daiichi Sankyo for the treatment and secondary prevention of AIS and acute PE [52,53]. It showed high selectivity for CPU over CPN (510.000-fold) and was 10-fold more potent than PTCI [165]. During preclinical assessment in a TF-induced rat micro-thrombosis model, it reduced existing fibrin clots in the lung after IV administration. Both oral and IV administration stimulated endogenous and rtPA-mediated fibrinolysis in this rat model without prolongation of the tail bleeding time. The first-in-man trials of IV and orally administered DS-1040 confirmed the efficacy and safety of the novel CPU inhibitor. DS-1040 was well tolerated by both young and elderly healthy volunteers and CPU activity decreased dose-dependently. After IV administration, the inhibitor had a long terminal half-life of 1.5 days and up to 80% could be recovered unchanged in the urine. As could be expected, endogenous fibrinolysis assessed by D-dimer determination was found to be increased in some individuals. Interestingly, an increase in bleeding time—although not clinically relevant—was observed in some young individuals [52]. Administration of DS-1040 showed no clinically significant bleeding or variations in coagulation markers. Equally, after oral administration, concentration-dependent inhibition of CPU activity was seen and a similar terminal half-life was observed as after IV administration, but only 10% of the compound could be recovered unchanged in urine. To evaluate pharmacokinetic (PK) interactions between DS-1040 and standard-of-care agents, a co-administration study with aspirin, clopidogrel, and enoxaparin has been performed (NCT02071004 and NCT02560688). The combination of DS-1040 with either of three compounds was well tolerated and did not cause severe adverse events (AEs) or increased bleeding risk. Some mild AEs were observed after administration of the inhibitor, but these were not different compared to the ones observed after administration of the established drugs. Furthermore, concomitant administration of a single IV dose of DS-1040 with oral clopidogrel or i.m. enoxaparin did not result in measurable PK drug-drug interactions [166]. Two phase Ib/II clinical trials evaluating the safety, tolerability and PK/PD of DS-1040 in subjects with acute ischemic stroke (ASSENT; NCT02586233 and NCT03198715) and acute submassive pulmonary embolism (NCT02923115) respectively have recently been completed. Not all results of the ASSENT trial have been published yet, but DS-1040 did not show significant improvement in patients with pulmonary embolism compared to placebo when intravenously administered in co-therapy with standard of care anticoagulation [174,175,176]. Recently, the development of DS-1040 has been discontinued [177].

S62798, a potent CPU inhibitor designed by Servier, demonstrated to have a favorable safety profile and a linear PK. The structure of S62798 has not been disclosed. All doses of S62798 were well-tolerated, without relevant adverse events. A rapid and dose-dependent inhibition of CPU in three different pharmacodynamic assays (ex vivo CPU inhibition and two variants of the in vitro clot lysis assay) was observed in all treated groups [95]. In a mouse model of pulmonary, thromboembolism, S62798 treatment, alone or associated with heparin, accelerated clot degradation by potentiating endogenous fibrinolysis and decreasing pulmonary fibrin deposition [164]. These promising data made it worthwhile to pursue clinical development for the treatment of thromboembolic diseases [95].

Berlex Biosciences developed BX528 [(S)-2-[3-(aminomethyl)phenyl]-3-{hydroxyl [(R)-2-methyl-1-{[(3-phenylpropyl) sulfonyl] amino propyl] phosphoryl propanoic acid], a potent CPU inhibitor with IC_50_ values in the low nanomolar range that showed good selectivity against other carboxypeptidases including CPN (>3500-fold) but limited selectivity against pancreatic CPB (12-fold). In an in vivo complement activation model in guinea pigs, only a minimal inhibition of plasma CPN activity was observed. Intravenous administration of BX528 enhanced rtPA-induced thrombolysis in a model of femoral artery occlusion in rats (laser-induced thrombosis) and dogs (10% FeCl_2_) when administered on top of a low dose of rtPA. Without rtPA, the inhibitor was not able to increase reperfusion in these models. In a rat disseminated intravascular coagulation (DIC) model, inhibition of CPU by BX528 attenuated LPS-induced resistance to endogenous fibrinolysis [157]. Hemodynamic alterations or influence on bleeding time was not observed in the presence of BX528 in all animal models tested [157,158]. The efficacy and safety of BX528 are encouraging, but oral bioavailability in rats was only 1% [158]. Further clinical development of this compound has not been reported.

EF6265 [(S)-7-amino-2-[[[(R)-2-methyl-1-(3-phenylpropanoylamino)propyl]hydroxy-phosphinoyl] methyl]heptanoic acid] was developed by Meiji Seika Kaisha (Tokyo, Japan) as an intravenously administered inhibitor of CPU. Systemic injection of EF6265 could efficiently lyse TF-induced microthrombi in a rat micro-thrombosis model while maintaining a low risk of bleeding [150]. In 2006, EF6265 was acquired by MediciNova and was named MN-462. So far, no progression into clinical development of EF6265/MN-462 has been reported. Preclinical results of EF6265 displaying a protective profile (reduced fibrin depositions in kidney and liver and decreased markers of organ dysfunction) against sepsis-induced organ dysfunction in rats have been reported by Muto et al. [151]. Recently, EF6265 was used as a lead for the optimization of novel CPU inhibiting compounds. A sulfur-containing lysine analog, DD2 [7-Amino-2-(sulfanylmethyl)heptanoic acid] and selenium-containing inhibitors have been described [145,178]. With an oral absorption of 30%, both oral and intravenous administration of DD2 enhanced endogenous fibrinolysis and reduced thrombi in a TF-induced micro-thrombosis model [178]. In the selenium-containing series, especially compound 12 [2,2′-(Diselane-1,2-diyldimethanediyl)bis[3-(6-amino-5-chloropyridin-3-yl)propanoic Acid]] was promising for further development with IC_50_ values in the low nanomolar range and excellent selectivity for CPU over CPN [145]. Whether DD2 and compound 12 are in further development has not been disclosed yet.

Researchers from Merck developed imidazole acetic acid inhibitors of CPU. Compound 10j [(-) 3-(6-aminopyridin-3-yl)-2-(1-(2-isopentyl-1H-imidazol-4-yl)propanoic acid dihydrochloride] described in [154] (also denoted compound (-)-8 [146]) was tested in several animal models including an African green monkey model of vascular injury where it showed efficacy in an ex vivo clot lysis assay. Compound 10j demonstrated oral bioavailability, had an IC_50_ of 2 nM, and a specificity vs. CPN of >25,000-fold but had a short terminal half-life. It prolonged the time-to-occlusion in the jugular vein in a dose-dependent manner without a physiologically meaningful difference in bleeding time [154,155]. Besides the characterization of compound 10j in some preclinical models, no further development has been disclosed.

The development of a number of these small molecule inhibitors from various manufacturers has been discontinued for various reasons, including the lack of oral bioavailability of the compound, the absence of superiority versus standard treatment or the challenging clinical trial set-up in complex acute pathologies such as stroke.

In the last 15 years, several novel antibodies and nanobodies have been developed to target human, rat, or murine CPU [179,180,181,182]. Direct CPU inhibition was obtained through monoclonal antibodies or nanobodies that block the catalytic site or that destabilize CPU [179]. Besides these highly specific, direct inhibitors of active CPU, antibodies that selectively impair the activation of proCPU by either thrombin-thrombomodulin or plasmin have also been characterized [179,180,182]. These antibodies and nanobodies accelerated in vitro clot lysis and have been extensively used as profibrinolytic tools and to further unravel (patho)physiologic activation and inactivation of proCPU [181,183,184]. Noteworthy, some antibodies and nanobodies have been reported to stimulate the intrinsic carboxypeptidase activity of proCPU in in vitro clot lysis models which was explained by the translocation of the activation peptide, making the catalytic cleft accessible for larger substrates such as C-terminal lysines on partially degraded fibrin [180,183]. Hendrickx et al. generated and characterized a nanobody against rat CPU that was evaluated in a mouse model of TF-induced thromboembolism. The nanobody significantly decreased fibrin depositions in the lungs in the absence of exogenously administered rtPA and enhanced the fibrinolytic efficacy of rtPA in co-administration [185]. Vercauteren and coworkers generated MA-TCK26D6, a monoclonal antibody (mAb) raised against human proCPU that modulates proCPU activation by thrombin and plasmin but not by the physiologically most relevant activator thrombin-thrombomodulin. MA-TCK26D6 demonstrated strong profibrinolytic effects in vitro and in an in vivo mouse thromboembolism model [184]. CPU activity was also inhibited by MA-TCK26D6 and another plasmin-specific CPU mAb (MA-TCK11A9), but this effect was substrate dependent as it interfered with the binding of CPU to fibrin but did not inhibit the anti-inflammatory activity of CPU on osteopontin and C5a [186]. Recently, Denorme et al. demonstrated that the combined use of MA-TCK26D6 and MA-33H1F7, a mAb that inactivates PAI-1, was protective in a mouse model of cerebral ischemia in mice [187]. Wyseure et al. fused MA-TCK26D6 with MA-33H1F7 into an innovative, bispecific diabody (Db-TCK26D6 × 33H1F7) that showed promising thromboprophylactic effects in a mouse model of venous thromboembolism. Also in two stroke models, a transient middle cerebral artery occlusion model (MCAO) and a thrombin-induced MCAO model, the diabody reduced lesion volume markedly. Its effect even exceeded the effect of 10 mg/kg rtPA in the thrombin-induced MCAO model. In the transient MCAO model, fibrin(ogen) deposition was also reduced which was associated with improved neurologic and motor outcome [188]. The diabody was recently patented [189].

Noteworthy, some competitive LMW inhibitors and nanobodies exhibit an ambiguous activity in in vitro clot lysis experiments. As expected at high concentrations, these inhibitors enhance fibrinolysis, but paradoxically, at very low concentrations, a small prolongation of the clot lysis time can be observed [181,190,191]. The observed prolongation of the clot lysis time is attributed to the appearance of two CPU pools when proCPU is activated in the presence of low concentrations of a CPU inhibitor. One pool of “circulating” free CPU and one inhibitor-bound pool. Free CPU is susceptible to rapid inactivation due to its intrinsic thermal instability, whereas inhibitor-bound CPU is stabilized by the inhibitor and thus protected from decay [190,191]. The two pools are in constant equilibrium and when free CPU is inactivated, the free CPU pool is replenished by CPU that is released from the CPU-inhibitor complex [190,191]. This phenomenon only occurs when not all circulating CPU is bound to the inhibitor, thus when the CPU concentration is high relative to the inhibitor concentration.

Overall, the currently evaluated inhibitors show an excellent safety profile and issues with selectivity towards CPN seem to be overcome. Selectivity against pancreatic CPB is obtained with antibodies targeting CPU, but remains difficult to achieve with LMW inhibitors. However, little risk for severe intestinal malabsorption is anticipated with transient inhibition of pancreatic CPB since other digestive proteases will still be present in the gastrointestinal system. Furthermore, some of the LMW compounds display excellent oral bioavailability were others, as well as antibodies, can only be administered intravenously. In acute settings such as AIS, an intravenous compound is preferred over oral administration given the fact that the latter results in a slower increase of plasma levels and is not possible in unconscious patients or patients facing difficulties with swallowing. However, in the setting of the prevention of recurrent stroke, or even in primary prevention, an orally administered compound is desirable. Although, if the terminal half-life of the inhibitor allows a less intense dosing regimen, a subcutaneous, i.m., or even an IV administered compound may be considered as well. As for antibody inhibitors, other major drawbacks are the risk of immunogenicity and the significantly higher production time and cost compared to LMW inhibitors [192]. Moreover, questions have also been raised about the ability of mAbs to penetrate a clot, as antibodies are significantly larger than LMW inhibitors, which might limit its applications in vivo. In this point of view, nanobodies—the smallest naturally-occurring antigen-binding antibody fragments—are of particular interest. Nanobodies are ten times smaller than conventional mAbs and display an increased clot penetration in an in vitro clot lysis model as demonstrated by Buelens and colleagues [182]. Furthermore, nanobodies combine several advantages of LMW compounds and mAbs such as high solubility, stability, low immunogenicity, and high affinity towards their targets, but they are still costly and time-consuming to manufacture [181,192]. A major drawback of the inhibitory nanobodies is the lack of species cross-reactivity between nanobodies raised against mouse and rat or human (pro)CPU which might hamper the translation from bench to bedside.

The ability of CPU to counteract efficient plasmin formation makes its inhibition an appealing strategy for the treatment of venous and arterial thrombotic disorders. In vitro evidence for the importance of the CPU pathway is provided by studies in which it is shown that the rate of fibrinolysis is significantly enhanced when either CPU is inhibited or proCPU is depleted [11,111,191]. In addition, in vivo evidence of the potential of a CPU inhibitor to improve endogenous fibrinolysis and thrombolysis in either the presence or absence of exogenous rtPA has been evaluated in several animal models over the years. In a recent study, administration of the LMW inhibitor DS-1040 as such stimulated endogenous fibrinolysis in a TF-induced rat micro-thrombosis model, whereas reviews of *s*tudies evaluating the administration of other CPU inhibitors alone in different animal models up, showed somewhat contradictory results [144,165,170,193]. Despite, the inconclusive results of CPU inhibition in various animal models, the first-in-man trials and phase II trial of DS-1040 and AZD9684 respectively showed increased D-dimer, a marker of fibrinolytic activity, in several patients which points towards stimulation of endogenous fibrinolysis [105,122,151]. As for the approach of using CPU inhibition as an adjuvant to tPA-mediated thrombolysis, reviews by Willemse et al. and Foley et al. on this topic suggest that CPU deficiency/inhibition may improve the thrombolytic efficacy of tPA [101,110,147]. Moreover, in co-administration with rtPA PTCI reduced thrombus weight in rat models of venous or arterial thrombosis where PTCI alone was ineffective [194]. CPU inhibition alone or in combination with PAI-1 inhibition or rtPA administration was—depending on the LMW inhibitor, antibody or nanobody used—successful in the reduction of infarct size, increasing the reperfusion rate or decreasing fibrin(ogen) depositions in the brain of infarcted mice [163,187,188]. This was in contrast with the findings in several knockout models where *cpb2* knockout mice did not show significant differences in infarct volume, functional outcome, or fibrin(ogen) in a tMCAO model [195]. Another study even showed an aggravation of infarct size after rtPA administration in a *cpb2* knockout [196]. Bleeding complications related to CPU inhibition have neither been reported in *cpb2* knockout mice nor after administration of CPU inhibitors. Westbury et al. recently showed that hemostasis was not significantly impacted in individuals with a partial proCPU deficiency, which underscores the potential of pharmacological inhibition of CPU for the treatment of thromboembolic diseases and encourages that this therapeutic strategy will unlikely result in bleeding complications [197,198]. However, data need to be interpreted with caution as only limited activation of proCPU (1–2%) can be enough to exert its antifibrinolytic effects.

For both approaches—CPU inhibitor alone and combined administration with tPA—it is important to bear in mind that the type of inhibitor and thrombosis model, but also interspecies differences and the timing of inhibitor administration (before or after thrombus induction) are determinants of the in vivo profibrinolytic efficiency of a CPU inhibitor. Moreover, whether or not an apparent effect of a CPU inhibitor alone can be observed also depends on the magnitude of the thrombotic stimulus that was applied [199].

## 6. Conclusions

As a molecular link between coagulation and fibrinolysis, CPU has gained interest as both a potential risk factor for thromboembolic diseases, as well as an attractive target in the treatment of these diseases. Today, there is a rationale that high proCPU levels carry a mild risk factor for the development of venous thrombosis, whereas results are inconclusive when it comes to arterial thrombosis. In addition, there is no consensus yet on the role of the Thr/Ile325 polymorphism as a risk factor in thromboembolic diseases in general. On the other hand, it has become clear that quantifying the extent of proCPU activation (e.g., by measurement of CPU activity or consecutive proCPU determinations) is a valuable tool that can help to broaden the understanding of the in vivo role of the CPU pathway during the acute stage of thromboembolic diseases. Furthermore, several CPU inhibitors have been developed and were tested in animal models of thromboembolic diseases with varying success rates. Some inhibitors have made it to clinical development and proved to be safe and well-tolerated in healthy volunteers.

## Figures and Tables

**Table 1:** Overview of in-house developed assays and commercially available quantification kits for the determination of different forms of the *CPB2* gene product in human plasma.

Assay Type		Interference or Influence of					Additional Information	Used in
		Thr/Ile325	CPN	ProCPU	CPU	CPUi		
**ProCPU measurement**								
Activity *	Wang et al. 1994 [26]	No	Yes	-	Yes	No	Hip-Arg with detection by RP-HPLC	
	Mosnier et al. 1998 [11]	No	Yes	-	Yes	No	Hip-Arg with colorimetric detection	[27,28,29]
	Schatteman et al. 1999 [30]	No	Yes	-	Yes	No	Hip-Arg with detection by RP-HPLC	[31,32,33]
	Schatteman et al. 2001 [34]	No	Yes	-	Yes	No	*p*-OH-Hip-Arg with colorimetric detection	[35]
	Heylen et al. 2010 [9]	No	No	-	Yes	No	Bz-*o*-cyano-Phe-Arg with detection by RP-HPLC; Not very sensitive to influence of hemolysis	[36]
	STA-Stachrom^®^ TAFI (Diagnostica Stago)	No	No	-	Yes	No	Azoformyl–AA2—AA1 (Patent: SERBIO PCT/FR 02/02376) with colorimetric detection	[37]
	Actichrome^®^ TAFI (American Diagnostica)	No	No	-	Yes	No	Chromogenic substrate with colorimetric detection; No longer marketed	[38,39,40,41,42,43,44]
	Pefakit^®^ TAFI (Pentapharm)	No	No	-	Yes	No	Synthetic substrate with colorimetric detection	[45,46,47,48,49,50,51,52,53]
ELISA	Mosnier et al. 1998 [11]	Unk.	No	-	Unk.	Unk.	Murine monoclonal capture and rabbit polyclonal detection antibody	[54,55]
	van Tilburg et al. 2000 [56]	No	No	-	Yes	Yes	Electroimmunoassay	[56,57,58,59,60]
	Strömqvist et al. 2001 [13]	Unk.	No	-	No	No	No reaction with plasma of other species (guinea pig, rat, dog, pig, hamster)	
	Ceresa et al. 2006 [12]	No **	No	-	No	No	Monoclonal capture and detection antibody	[58,59,61,62,63]
	VisuLize^®^ TAFI (Affinity Biologicals)	Yes	No	-	Yes	Yes	Sheep polyclonal capture and detection antibody; Marketed by Milan Analytica	[40,41,42,43,64,65,66,67,68,69,70]
	Imuclone^®^ TAFI (American Diagnostica)	Yes	No	-	No	No		[71,72,73,74,75]
	Asserachrom^®^ TAFI-1B1 (Diagnostica Stago)	No	No	-	No	No	Previously marketed by Kordia Laboratory Supplies; No longer marketed	[51,76,77,78,79,80,81,82,83,84]
	Zymutest^®^ (Total) TAFI (Hyphen BioMed)	No	No	-	No	No	Previously marketed as Zymutest^®^ proTAFI	[38,85,86,87,88]
	Coalize^®^ TAFI (Chromogenix)	Unk.	No	-	Unk.	Unk.	Monoclonal capture and polyclonal detection antibody	[39,89]
**Activation peptide**								
ELISA	Ceresa et al. 2006 [12]	No ***	No	No	Yes	No	Monoclonal capture and detection antibody; Measures both AP and CPU	[58,59,61,63]
**Active CPU**								
Activity	Hendriks et al. 1989 [90]	No	Yes	No	-	No	*p*-OH-Hip-Arg with colorimetric detection and Hip-Arg with detection by RP-HPLC	
	Kim et al. 2008 [91]	No	No	No	-	No	Plasmin-modified fibrin is covalently bound to a quencher molecule and mixed with fluorescein-labeled plasminogen and the plasma sample. The rate of fluorescence increase detected by a plate reader reflects the amount of CPU present in the sample. High sensitive (LOD: 12 pM); Not affected by other hemostatic factors	
	Heylen et al. 2010 [92]	No	No	No	-	No	Bz-*o*-cyano-Phe-Arg with detection by RP-HPLC; High sensitive (LOD: 18 pM); Highly sensitive to influence of hemolysis	[93,94,95]
ELISA	Zymutest^®^ (Activatable) TAFI (Hyphen BioMed)	Yes					Previously marketed as Zymutest^®^ TAFI	[53,96,97]
Active & inactive CPU ****								
ELISA	Asserachrom TAFIa/ai (Diagnostica Stago)	No	No	No	-	-	Combined measurement of CPU and CPUi; Not sensitive to influence of hemolysis	[37,63]
	Imubind^®^ TAFIa/ai (Biomedica Diagnostics)	No	No	No	-	-	No longer marketed	[98]

This is a non-exhaustive list of methods that are available for the measurement of different forms of the *CPB2* gene product. Methods used in clinical studies that are discussed in this review (or derivative methods) were included in this table. * Requires quantitative activation of proCPU prior to measurement. Consecutive measurements can reveal proCPU consumption (ongoing CPU activation). ** T12D11/T30E5. *** T12D11/T18A8. **** Reflects past or ongoing CPU generation over a longer period. AA: amino acid; AP: activation peptide; CPN: carboxypeptidase N; CPU: carboxypeptidase U (TAFIa, CPB2); CPUi: inactivated CPU; ELISA: enzyme-linked immunosorbent assay; LOD: limit of detection; proCPU: procarboxypeptidase U (TAFI, proCPB2); RP-HPLC: reversed phase high pressure liquid chromatography; TAFI: thrombin-activatable fibrinolysis inhibitor (proCPU, proCPB2); Unk.: unknown.

**Table 2:** Studies investigating the role of proCPU plasma concentrations and proCPU gene (*CPB2* gene) polymorphism as risk factors for thromboembolic diseases.

**Venous Thrombosis**		
Arauz et al. 2018 [119]	No risk association in CVT cases (N = 113) relative to controls (N = 131) for the +1040C/T polymorphism or in haplotype analysis of the *CPB2* gene.	PCR
Orikaza et al. 2014 [120]	The +1040C/T polymorphism significantly increased the risk of CVT (N = 72) compared to VTE cases (N = 128) and controls (N = 134).	PCR
Tokgoz et al. 2013 [121]	In 59 patients with CVT and 100 healthy control subjects, the association between the +1040C/T polymorphism and CVT was investigated. Frequencies of polymorphic genotype and allele were similar in patients and controls and were not significant for CVT.	PCR
Meltzer et al. 2010 [45]	In a study involving 770 patients from the MEGA study (first DVT of the leg or first PE) and 743 controls, high proCPU levels were shown to be an independent risk factor for VTE.	Activity assay (Pentapharm)
Meltzer et al. 2010 [114]	The +1040C/T polymorphism was associated with both proCPU levels and increased risk for recurrent VTE in 474 patients diagnosed with first DVT.	PCR + In-house developed activity assay [56]
Verdu et al. 2008 [76]	It was found that the Thr/Thr genotype of the Thr/Ile325 polymorphism was associated with an increased risk of VTE.	PCR + ELISA (Diagnostica Stago)
Folkeringa et al. 2008 [47]	In a large cohort of thrombophilic families, no correlation was observed between high proCPU levels and the risk of venous or arterial thromboembolism.	Activity assay (Pentapharm)
Verdu et al. 2006 [122]	High proCPU levels (>90th percentile of the controls) increased the risk for future DVT 4-fold compared to patients with lower proCPU levels. 60 patients with previous DVT or PE and 62 controls were included in the study.	ELISA (Diagnostica Stago)
Martini et al. 2006 [60]	Thr/Ile325 polymorphism is associated with proCPU antigen levels in 471 patients with first DVT, but there was no association with increased risk for DVT.	PCR + In-house developed activity assay [56]
Zee et al. 2005 [123]	No evidence was provided for an association between six polymorphisms, including the +1040C/T polymorphism, in the *CPB2* gene and the risk for VTE.	PCR
Eichinger et al. 2004 [77]	Higher proCPU levels in patients with a previous first spontaneous VTE were associated with an almost 2-fold higher risk for VTE recurrence compared to patients with lower proCPU levels (N = 600 total study population).	ELISA (American Diagnostica)
Libourel et al. 2002 [115]	Symptomatic Factor V Leiden carriers (N = 17) had higher proCPU levels compared to asymptomatic carriers (N = 136). High levels of proCPU are a mild risk factor for VTE.	In-house developed activity assay [30]
Franco et al. 2001 [29]	A tendency towards protection against DVT was observed for a several *CPB2* gene polymorphisms, that paralleled the lower proCPU levels detected in carriers of these polymorphisms (N = 388) compared to controls (N = 388).	PCR + In-house developed ELISA [11]
Van Tilburg et al. 2000 [56]	ProCPU levels were similar in patients with a first episode of DVT (N = 474) compared to controls (N = 474). Although, there were more DVT patients than controls with high proCPU levels and high proCPU levels were associated with an increased risk for thrombosis.	In-house developed activity assay [56]
**Arterial thrombosis and coronary artery disease (CAD)**		
Isordia-Salas et al. 2019 [124]	Thr/Ile325 polymorphism in the *CPB2* gene was associated with an increased risk for STEMI (N = 244), but not for IIS (N = 250). Genotype and allele distribution were similar in IIS patients and controls (N = 244).	PCR
Rattanawan et al. 2018 [125]	The +1040C/T polymorphism was not associated with the severity of coronary artery stenosis.	PCR
Khalifa et al. 2012 [98]	ProCPU levels were not correlated with the pre-disposition of in-stent restenosis following coronary stenting in 37 patients with CAD.	CPU + CPUi ELISA (American Diagnostica)
Jood et al. 2012 [61]	No association between intact proCPU antigen and survival rate, nor reoccurrence of vascular events (recurrent stroke, transient ischemic attack or coronary event; N = 37) in ischemic stroke survivors (N = 517). Blood was collected 3 months after the index event. Two years after inclusion the survival rates and vascular events were assessed.	In-house developed ELISA [12]
De Bruijne et al. 2011 [59]	Increased levels of intact proCPU antigen are associated with an increased risk of premature peripheral arterial disease. Functional proCPU was not significantly higher in patients (N = 47) compared to controls (N = 141). Blood samples were collected 1–3 months after the event.	In-house developed ELISA [12] + In-house developed activity assay [126]
Kamal et al. 2011 [127]	Homozygous and heterozygous carriers of the Ile325 were more frequent in patients with AMI (N = 46) than in controls (N = 54).	PCR
Kozian et al. 2010 [128]	Homozygosity for the Ile325 allele of the Thr/Ile325 polymorphism was associated with the incidence of stroke and the age at onset of first stroke (N = 3300).	PCR
Tassies et al. 2009 [78]	ProCPU polymorphism Thr/Ile325 is related to the type of acute coronary syndrome (N = 248; total cohort). Homozygous 325Ile genotypes are less prevalent in patients with STEMI compared to NSTEMI patients.	ELISA (Diagnostica Stago) + Activity assay (American Diagnostica)
De Bruijne et al. 2009 [58]	In young patients with arterial thrombosis (N = 327), the distribution of the Thr/Ile325 SNP was compared to healthy controls (N = 332). In homozygous carriers of the Ile325 allele lower proCPU levels were observed together with a decreased risk of arterial thrombosis compared to homozygous carriers of the Thr325 allele. In the same population total proCPU levels and proCPU activity levels did not differ from controls. Blood samples were collected 1–3 months after the event.	In-house developed ELISA [12] + In-house developed activity assay [126]
Tregouet et al. 2009 [79]	In a prospective study on patients with angiographically proven coronary artery disease none of the selected CPB2 gene polymorphisms, including Thr/Ile325, was associated with the occurrence of cardiovascular events (N = 1668). In the same study, the total proCPU antigen was associated with an increased risk of future cardiovascular death.	PCR + ELISA (Diagnostica Stago)
Meltzer et al. 2009 [46]	The +1040C/T SNP was not associated with myocardial infarction in men (N = 554) versus controls (N = 643). Also, patients with proCPU levels in the first quartile (lowest levels) display an increased risk of a first myocardial infarction compared to patients with proCPU levels in the fourth quartile. The time between blood sampling after the infarct ranged from 88 days to 5.8 years with a median of 2.6 years.	PCR + Activity assay (Pentapharm)
Biswas et al. 2008 [80]	No association was detected between 16 single nucleotide polymorphisms (of which the Thr/Ile325 polymorphism was one) and the risk of cardio-embolic stroke (N = 120). Also, significantly higher proCPU antigen levels were observed in patients with acute onset non-cardioembolic stroke (N = 120) compared to normal individuals (N = 120). Blood samples were collected within 10 days of the stroke and at 3-month follow-up.	ELISA (Diagnostica Stago)
Ladenvall et al. 2007 [62]	No association was detected between 11 single nucleotide polymorphisms (including Thr/Ile325 SNP) and overall ischemic stroke risk (N = 600). In addition, increased levels of intact proCPU are found in ischemic stroke patients compared to controls (N = 600). An independent association was found with large vessel disease, cryptogenic stroke and acute-phase small vessel disease subtypes. Increased proCPU levels do not reflect an acute phase response. Blood sampling was conducted within 10 days of the stroke and at 3-months follow-up.	PCR + In-house developed ELISA [12]
Fernandez-Cadenas et al. 2007 [129]	Ile/Ile homozygosity for the Thr/Ile325 polymorphism was associated with lower rates of recanalization after rtPA infusion in ischemic stroke patients (N = 139).	PCR
Cruden et al. 2006 [38]	Plasma proCPU does not predict reperfusion in patients receiving thrombolytic therapy for acute STEMI (N = 110). Blood was collected prior to administration of thrombolytic therapy.	ELISA (Hyphen Biomed) + Activity assay (American Diagnostica)
Schroeder et al. 2006 [48]	Re-evaluation of the study in 2002 which was compromised by genotype-dependent artifacts. Significant associations between proCPU activity and cardiovascular risk factors as well as with coronary artery disease were found. ProCPU activity was higher in coronary artery disease patients (N = 338) than in controls (N = 158). Blood samples were collected during angiography.	Activity assay (Pentapharm)
Morange et al. 2005 [81]	No clear relation between coronary heart disease and six proCPU gene polymorphism was found (including Thr/Ile325). A total of 248 cases and 493 controls were used. And no significant association between proCPU levels and angina pectoris or hard coronary events was present after re-analysis of the PRIME data with an ELISA which was shown to be insensitive to proCPU genotype.	PCR + ELISA (Diagnostica Stago)
Leebeek et al. 2005 [130]	The proCPU genotype does not seem to predict the risk of ischemic stroke. No difference was found between patients (N = 124) and controls (N = 125) with respect to the distribution of the +1040C/T polymorphism. Also, increased proCPU levels, resulting in decreased fibrinolysis, are associated with an increased risk of first ischemic stroke (ischemic stroke N = 124 vs. controls N = 125). As demonstrated by the persisting elevated proCPU levels three months after the stroke, the increase in functional proCPU levels is not caused by an acute phase reaction. Blood collection between 7 and 14 days after the stroke, second blood collection in a subgroup (N = 36) three months after the stroke.	PCR + In-house developed functional TAFI clot lysis assay [126]
Lisowski et al. 2005 [40]	ProCPU levels were significantly higher 7 days after elective CABG in 45 stabile angina pectoris patients with confirmed CAD compared to controls (N = 33).	ELISA (Affinity Biologicals) + Activity assay (American Diagnostica)
Kim et al. 2005 [86]	No difference was seen in proCPU levels in acute ischemic stroke patients with (N = 30) or without successful recanalization (N = 13). Blood samples were collected on admission.	ELISA (Hyphen Biomed)
Santamaria et al. 2004 [131]	ProCPU levels tended to be higher in patients with acute CAD (N = 174) than in controls (N = 211). Blood samples were collected at least six months after the acute episode.	In-house developed activity assay [11]
Segev et al. 2004 [72]	It was shown that the proCPU antigen level is strongly determined by the Thr/Ile325 polymorphism in patients with stable angina pectoris (N = 159). The T/T genotype was the least prevalent and associated with the lowest proCPU levels and the lowest rate of angiographic restenosis in this population.	PCR + ELISA (American Diagnostica)
Akatsu et al. 2004 [132]	In 253 patients with confirmed neuropathology that died during hospitalization, no statistical correlation between the Thr/Ile325 polymorphisms and risk for cerebral infarction was found.	PCR
Morange et al. 2003 [85] Re-evaluated in 2005	In France, proCPU levels were significantly higher in men who subsequently developed angina pectoris (N = 81) than in their controls (N = 81), whereas no difference was observed between cases (N = 62) and controls (N = 124) in Northern Ireland.	PCR + ELISA (Hyphen Biomed)
Brouwers et al. 2003 [65]	No significant association between the Thr/Ile325 polymorphism was found in non-refractory patients (N = 133) compared to refractory patients (N = 76) with unstable angina pectoris. Higher proCPU levels in patients with non-refractory unstable angina pectoris (N = 133) than in refractory patients (N = 76). Blood samples were obtained on admission.	PCR + ELISA (Affinity Biologicals)
Zorio et al. 2003 [39]	No difference according to the Thr/Ile325 polymorphism between young patients with myocardial infarction (N = 127) and controls (N = 99). Patients had higher plasma proCPU activity levels, but lower proCPU antigen in comparison to controls. Blood sample was collected at least 3 months after the myocardial infarction.	PCR + ELISA (Chromogenix) + Activity assay (American Diagnostica)
Lau et al. 2003 [71]	Higher preprocedural proCPU plasma levels in patients with restenosis. ProCPU plasma levels correlated with 6-month % diameter stenosis after percutaneous coronary intervention (N = 159).	ELISA (American Diagnostica)
Juhan-Vague et al. 2003 [67]	No difference in proCPU plasma concentration in men who subsequently suffered from myocardial infarction or coronary death (N = 159) when compared with their controls (N = 317). Prospective study.	ELISA (Milan Analytica)
Santamaria et al. 2003 [28]	Higher proCPU plasma levels in ischemic stroke patients (N = 114) than in healthy controls (N = 150). Blood samples collected at least one month after the acute thrombotic episode	In-house developed activity assay [11]
Juhan-Vague et al. 2002 [68]	Patients who suffered from AMI (N = 598) showed lower plasma proCPU antigen values versus controls (N = 653). Blood samples were collected 3–5 months after myocardial infarction.	PCR + ELISA (Milan Analytica)
Morange et al. 2002 [133]	The Thr/Ile325 polymorphism does not influence the risk of MI in white male patients younger than 60 years who survived a first MI (N = 533) and male controls of the same age (N = 575).	PCR
Schroeder et al. 2002 [66] Re-evaluated in 2006	Higher proCPU antigen levels in coronary artery disease patients (N = 362) compared to controls with angiographically verified normal coronary vessels (N = 134). The difference was more prominent in intracoronary than in venous blood samples. Blood samples were collected during angiography.	ELISA (Milan Analytica)
Silveira et al. 2000 [31]	Higher proCPU plasma concentration in men requiring coronary artery bypass grafting because of stable angina pectoris (N = 110) than in controls (N = 56). Blood samples were collected preoperative.	In-house developed activity assay [30]

Table based upon earlier overviews published by Leurs et al. [8] and Heylen et al. [18], complemented with additional and new reports. Meta-analyses were not included. AMI: acute myocardial infarction; CAD: coronary artery disease; CVT: cerebral venous thrombosis; DVT: deep vein thrombosis; ELISA: enzyme-linked immunosorbent assay; IIS: idiopathic ischemic stroke; NSTEMI: non-ST-elevation myocardial infarction; PCR: polymerase chain reaction; proCPU: procarboxypeptidase U (TAFI, proCPB2); rtPA: recombinant tissue-type plasminogen activator; SNP: single nucleotide polymorphism; STEMI: ST-elevation myocardial infarction; VTE: venous thromboembolism.

**Table 3:** Studies investigating the role of CPU and/or CPUi as diagnostic or prognostic biomarkers for thromboembolic diseases during the acute phase.

Arterial Thrombosis and Coronary Artery Disease (CAD)		
Alessi et al. 2016 [37]	CPU/CPUi levels were monitored in 109 patients with ischemic stroke, with 41 receiving rtPA. Blood samples were collected post-admission/post-thrombolysis up to day 90 at 8 different time points. AIS patients had higher levels of CPU/CPUi at admission in comparison with the control population. In thrombolysed patients, an increase in CPU/CPUi was observed at the end of thrombolytic therapy, which lasted up to 4 h. In the non-thrombolysed group, CPU/CPUi levels did not differ over time. Both in thrombolysed as in non-thrombolysed patients, higher CPU/CPUi levels were associated with a more severe stroke and unfavorable outcome. No details available on sample collection and the use of inhibitors to prevent ex vivo proCPU activation.	CPU + CPUi ELISA (Diagnostica Stago)
Leenaerts et al. 2015 [93]	During the acute phase of myocardial infarction, CPU activity levels are higher in patients with AMI (N = 45) than in controls (N = 42). No association was found between CPU activity and AMI type (NSTEMI vs. STEMI). Intracoronary samples contained higher CPU levels than peripheral samples, indicating increased local CPU generation. Blood samples were collected at the start coronary catheterization. Blood collected in prechilled tubes containing sodium citrate, PPACK and aprotinin. Samples were immediately placed on ice after collection.	In-house developed activity assay [92]
Brouns et al. 2009 [33]	In patients with ischemic stroke (N = 12) receiving thrombolytic therapy, the amount of CPU generated is associated with evolution of the neurological deficit as well as with achieved recanalization. Blood samples were taken at 6–9 different time points before, during and after thrombolytic therapy. Blood collected in prechilled tubes containing sodium citrate, PPACK and aprotinin. Samples were immediately placed on ice after collection.	In-house developed activity assay [139]
Willemse et al. 2008 [99]	CPU activity is induced during therapeutic thrombolysis of patients with acute ischemic stroke (N = 8). Blood samples were collected at six to nine time points before, during and after thrombolysis. Blood collected in prechilled tubes containing sodium citrate, PPACK and aprotinin. Samples were immediately placed on ice after collection.	In-house developed activity assay [139]

AIS: acute ischemic stroke; AMI: acute myocardial infarction; CAD: coronary artery disease; CPU: carboxypeptidase U; CPUi: inactivated CPU; ELISA: enzyme-linked immunosorbent assay; NSTEMI: non-ST-elevation myocardial infarction; PCR: polymerase chain reaction; proCPU: procarboxypeptidase U (TAFI, proCPB2); rtPA: recombinant tissue-type plasminogen activator; STEMI: ST-elevation myocardial infarction.

**Table 4:** Filed patents for CPU inhibitors with their corresponding lead compounds and highest development status reported.

Company	Patents	Chemical Structure	Lead Compound	Highest Development Status Reported	Reference
AstraZeneca	WO/2000/66550 WO/2000/066557 WO/2000/066152 WO/2003/106420 WO/2003/027128	Phosphor and/or thiol-containing compounds	AZD9684	Phase II completed; development halted	[36,147,148]
	WO/2005/039617	Cyclic anabaenopeptin-type peptides	Unknown	Discovery	
	Unknown	Conformationally restricted imidazole derivatives	Compounds 7 and 12	Preclinical	[149]
Meiji Seika Kaisha/ MediciNova *	WO/2001/19836	Phosphonic and phosphinic acid derivatives	EF6265/MN-462 *	Preclinical	[150,151]
Pfizer	WO/2002/14285 WO/2003/061652 WO/2003/061653	Imidazole propanoic acid derivatives	UK-396082	Phase I completed, development halted	[152,153]
Merck	WO/2003/013526	Imidazole acetic acid derivatives	Compound 10j/ Compound (-)-8	Preclinical	[154,155]
Berlex Biosciences (Bayer)	WO/2003/080631 WO/2006/041808	Aryl guanidinic and mercaptopropionic acid derivatives	BX 528	Preclinical	[156,157,158]
Sanofi	WO/2005/105781 WO/2007/045339 WO/2010/130718 WO/2013/076178 WO/2013/076179 WO/2008/067909	Imidazole, urea & sulfamide containing compounds	SAR104772 SAR126119	Phase I completed Phase II initiated in 2013	[159]
Sanofi	WO/2009/146802 WO/2014/198620	Macrocyclic urea derivatives	Unknown	Preclinical	[160,161,162]
		Unknown	FFC.HTZ4.059	Preclinical	[163]
Servier	WO/2010/149888	2-mercapto-cyclopentane carboxylic acid compounds	Unknown	Discovery	
	WO/2017/121969	Phosphinane & azaphosphinane derivatives	S62798	Phase I completed	[95,164]
Taisho Pharmaceutical	WO/2011/034215	Dihydroimidazo-quinoline compounds	Unknown	Discovery	
Daiichi Sankyo	WO/2011/115064 WO/2011/115065 WO/2013/039202	Cycloalkyl-substituted imidazole, cyclopropanecarboxylic acid, and acrylic acid derivatives	DS-1040	Phase Ib/II completed; development halted	[52,53,165,166]
	WO/2016/204239 WO/2016/104678	Combinations of a CPU inhibitor with other compounds	DS-1040 + rtPA DS-1040 + Edoxaban		
	WO/2017/170460	CPU inhibitor application in inflammatory bowel disease			

* EF6265 was obtained by MediciNova from MSK in 2006 and renamed MN-462.

## Data Availability

Not applicable.
